# Accuracy of image-free navigation in intraoperative leg length change from total hip arthroplasty using evaluations from 2D and 3D measurements

**DOI:** 10.1186/s12891-021-04906-1

**Published:** 2021-12-06

**Authors:** Shine Tone, Masahiro Hasegawa, Yohei Naito, Hiroki Wakabayashi, Akihiro Sudo

**Affiliations:** Department of Orthopaedic Surgery, Mie University, Graduate School of Medicine, 2-174 Edobashi, Tsu, Mie 514-8507 Japan

**Keywords:** Image-free navigation, Leg length change, Total hip arthroplasty, 2D measurement, 3D measurement

## Abstract

**Background:**

Leg length discrepancy is one of the most common problems after total hip arthroplasty (THA). The aim of this study was to investigate the accuracy of image-free navigation in intraoperative leg length change (LLC) using evaluations from anteroposterior radiographs (2D measurement) and 3D bone models using CT data (3D measurement).

**Methods:**

One hundred THAs with cementless cups and stems were performed using an image-free navigation system in our hospital. We evaluated the accuracy of image-free navigation based on LLC from 2D and 3D measurements. Furthermore, we also investigated error in absolute value and correlations between 2D and 3D measurements in LLC.

**Results:**

The accuracy of image-free navigation based on 2D measurement was 94% within 5 mm and 76% within 3 mm. The accuracy of image-free navigation based on 3D measurement was 92% within 5 mm and 81% within 3 mm. The error in absolute value in LLC between 2D and 3D measurements was 1.7 ± 1.4 mm (range, 0 to 6 mm). A strong correlation was observed between 2D and 3D measurements in the LLC.

**Conclusions:**

In the present study, good accuracy of image-free navigation in intraoperative LLC was confirmed for both evaluation methods from 2D and 3D measurements. In addition, the error in absolute value in the LLC between 2D and 3D measurements was very small, and we observed a strong correlation between 2D and 3D measurements. Based on these results, evaluation of LLC from radiographs was considered sufficient if radiographs can be taken accurately.

## Background

Leg length discrepancy (LLD) is one of the most common problems after total hip arthroplasty (THA). LLD can be a cause of patient dissatisfaction because of ipsilateral gait disorder, knee pain, back pain and implant failure associated with inferior clinical outcomes [1–6]. Several methods have been reported for adjusting and equalizing leg length after THA [3, 7–12]. As one of these methods, use of computer navigation in THA has been increasing over the last decade. Many reports have described good accuracy for CT-based navigation and image-free navigation in terms of intraoperative leg length change (LLC) [13–18]. However, almost all those reports evaluated LLC using a two-dimensional (2D) measurement based on pre- and postoperative anteroposterior radiographs. In recent years, a three-dimensional (3D) method based on 3D bone models from computed tomography (CT) data has been used for preoperative and postoperative evaluation of total joint arthroplasty. Several reports have described that the evaluation using 3D method was more accurate than using 2D method for implant position and offset [19–21]. Therefore, we hypothesized that the 3D measurement could evaluate leg length change more accurate than the 2D measurement. No reports appear to have compared between the 2D and 3D measurements for the accuracy evaluation of image-free navigation. The aim of this study was to investigate the accuracy of image-free navigation in intraoperative LLC using evaluation methods based on 2D and 3D measurements.

## Methods

### Study design and patient selection

This retrospective case series was approved by the ethics committee of our institution (No. H2018–083). From November 2014, our institution performed 118 consecutive primary THAs with cementless cup and stems (Kyocera, Kyoto, Japan) using an image-free navigation system (Brain Lab; KICK Hip application 6.0, Helmstetten, Germany). We recorded preoperative variables, including age, sex, body mass index and primary diagnosis. For assessment of leg length, all included patients underwent pre- and postoperative radiographic and CT examinations. Postoperative radiography and CT examinations were performed at 2 weeks postoperatively. Exclusion criteria were incomplete operation record and inadequate pelvic x-rays and CT data. All preoperative and postoperative evaluations were performed by the author (ST), and all operations were performed by two senior surgeon (AS and MH).

### Surgical procedure

**Fig. 1:**
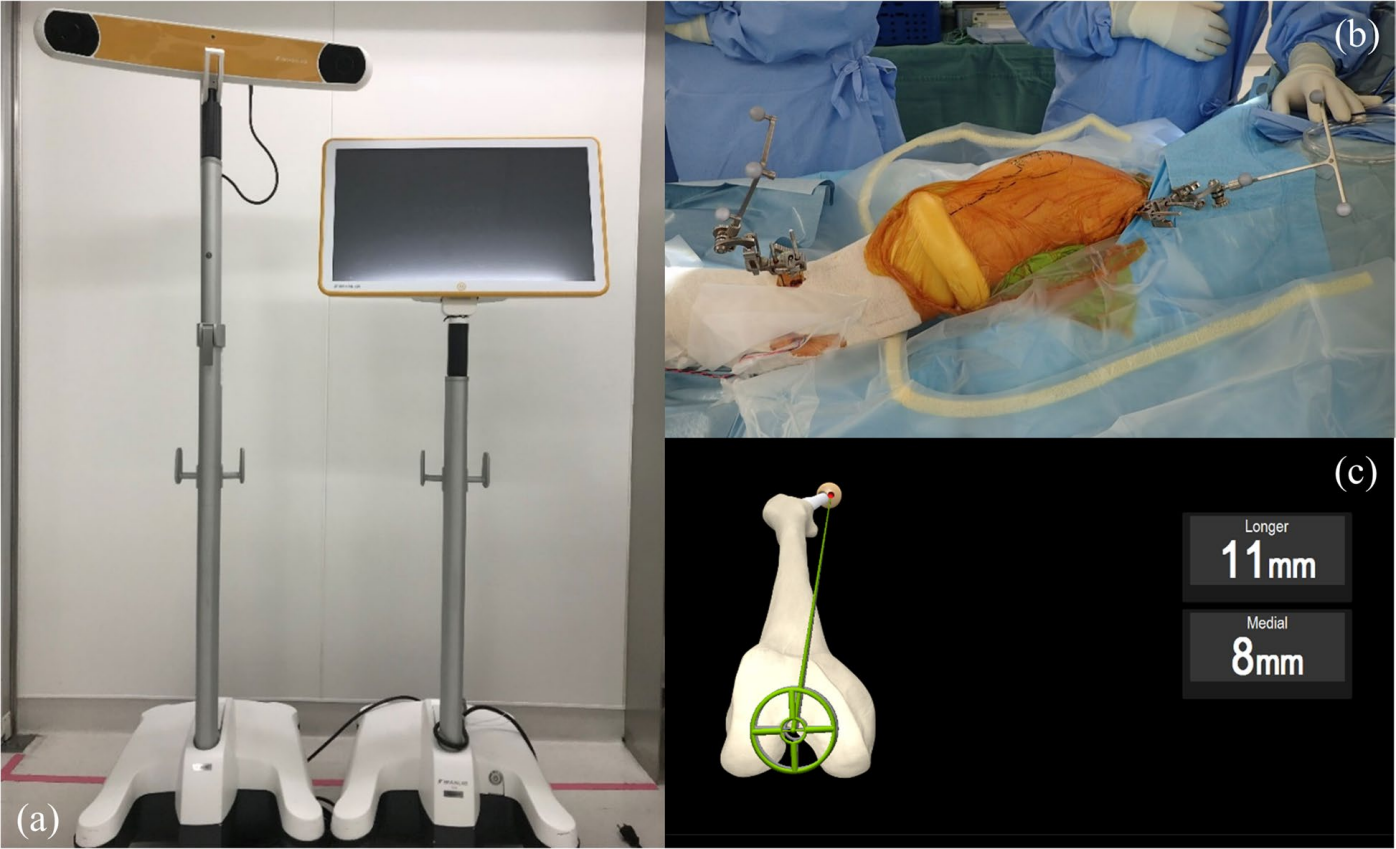
**a** The image-free navigation system (Brain Lab, KICK Hip application 6.0, Helmstetten, Germany). **b** Setting position of trackers on the pelvis and distal femur with two pins each. **c** Monitor image showing intraoperative leg length and offset values

For the THA procedure, we used a posterolateral approach in the lateral decubitus position. KICK Hip application system for THA is a non-image-based system that uses a virtual data model supplemented by intraoperative registration (Fig. 1a). The system required placement of trackers on the pelvis and distal femur (two pins each) before surgery (Fig. 1b). The reference frame used is the anterior pelvic plane, which is obtained by palpating bilateral anterior superior iliac spines (ASISs) with a special tracked palpation pointer registered to the computer. The femoral reference plane is formed by the piriformis fossa, medial and lateral epicondyles and ankle center. The femur position was registered to the software by holding the leg in a neutral extension position. After trial and final reconstructions, the femur is brought to the neutral position stored during leg alignment, so as to match the centers of the crosshairs displayed on the monitor. When these crosshairs are sufficiently aligned (within 5° of stored leg alignment), the active crosshair turns green. Intraoperative LLC was displayed after holding the leg steady for 2–3 s (Fig. 1c). Intraoperative LLC was defined as the amount of change from the leg length measured as the distance between the two trackers in the neutral position. All procedures were performed in accordance with relevant guidelines.

### Evaluation methods of leg length change

**Fig. 2:**
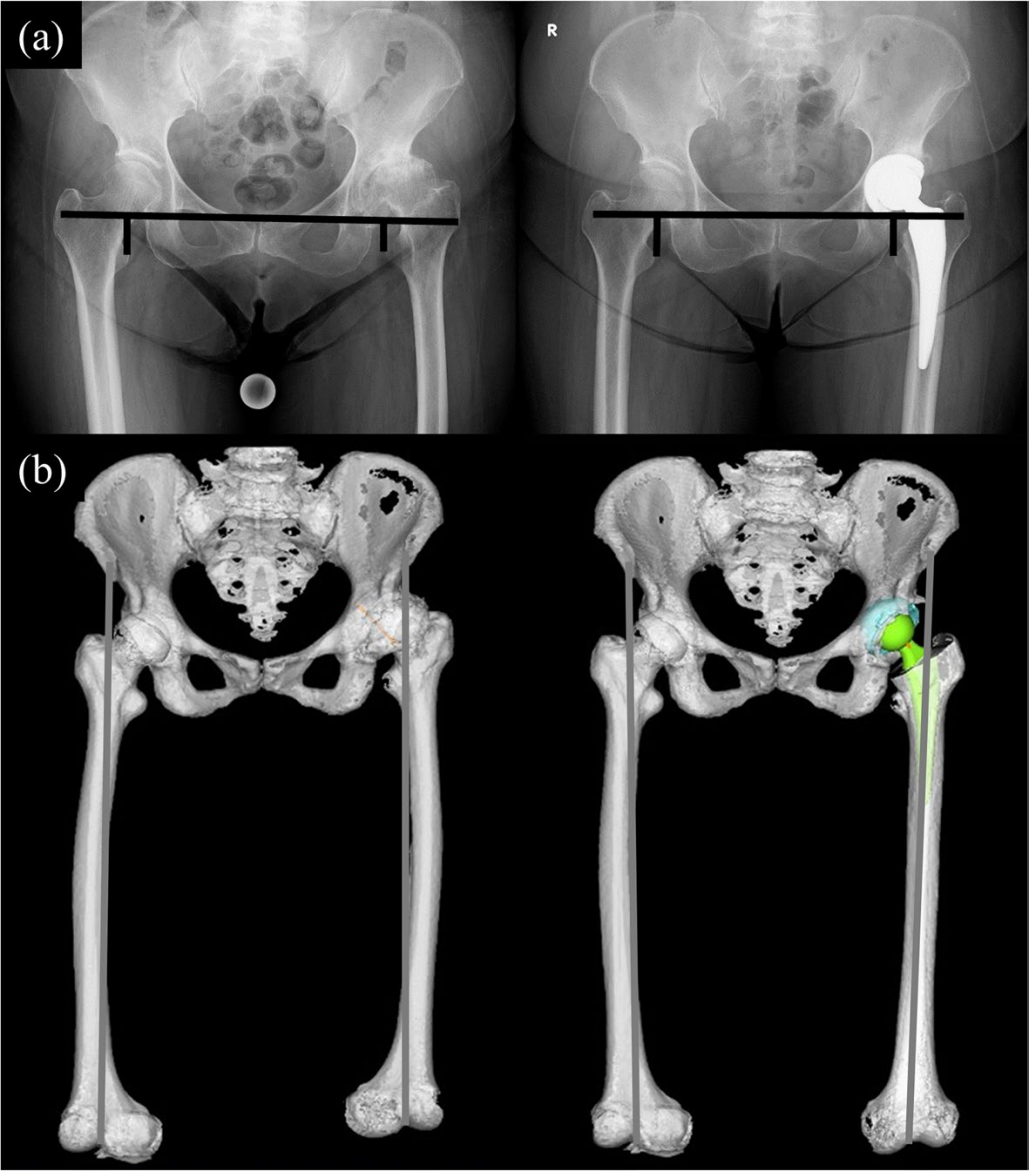
**a** Assessment of leg length discrepancy on the anteroposterior radiograph. Distance between the line connecting both tear drops and the medial apex of the lesser trochanter was measured. **b** Assessment of leg length discrepancy on the functional pelvic plane after repositioning using the 3D-Template system. Distance from the anterior superior iliac supine to the intercondylar fossa of femur was measured

The anteroposterior pelvic radiograph was performed in the standard manner with the patient supine with lower limbs placed in internal rotation and the big toes touching each other so that the patella was facing forward. Pre- and postoperative LLD on 2D measurement was measured as the distance between the horizontal line connecting both tear drops and the medial apex of the lesser trochanter in neutral position (Fig. 2a). LLC on 2D measurement was defined as the difference between pre- and postoperative LLD on 2D measurement. Helical CT providing images with a 1-mm slice interval from the ASIS to the knee was performed for all cases. Pre- and postoperative LLD on 3D measurement were measured in the functional pelvic plane after repositioning using the 3D-Template system (ZedHip; LEXI Co., Tokyo, Japan), then assessed as the distance from the ASIS to the intercondylar fossa of femur (Fig. 2b). The 3D-Template system was used to match pre- and postoperative CT digital images. LLC on 3D measurement was defined as the difference between pre- and postoperative LLD on 3D measurement. To evaluate the accuracy of image-free navigation based on 2D and 3D measurements, intraoperative LLC were compared with LLC on 2D and 3D measurements. We also investigated the error in absolute value and the correlation between 2D and 3D measurements of LLC.

### Statistical analysis

All statistical analyses were performed with EZR (Saitama Medical Center, Jichi Medical University, Saitama, Japan), a graphical user interface for R (The R Foundation for Statistical Computing, Vienna, Austria). Continuous data were analyzed using the nonparametric Wilcoxon signed-rank test and Spearman’s rank correlation coefficient. Values of *P* < 0.05 were considered significant. The reproducibility of 2D and 3D measurements was confirmed. For intra-observer reliability, each parameter was measured twice, on 20 hips, at an interval ≥ 4 weeks by one orthopedic surgeon (ST). For inter-observer reliability, two orthopedic surgeons (ST and YN) measured each parameter twice, on 20 hips, at an interval ≥ 4 weeks. Intra-class and inter-class correlation coefficient was calculated to analyse the variability between observers. Values of 0.81–1.00 indicated excellent correlation; 0.61–0.80, substantial correlation; 0.41–0.60, moderate correlation; 0.21–0.40, fair correlation; and 0.00–0.20, poor correlation.

## Results

One hundred patients with complete data sets were included for analysis. The patient demographics are shown in Table 1. The intra-class and inter-class correlation coefficients for the 2D measurement were 0.98 and 0.92, respectively. The intra-class and inter-class correlation coefficients for the 3D measurement were 0.97 and 0.94, respectively.

**Table 1:** demographic data

Variable	Results
Age (years)	67.9 ± 9.7
Gender	Male: 19
	Female: 81
Body mass index (kg/m^2^)	23.9 ± 4.5
Primary diagnosis	
Osteoarthritis	Crowe classification
	I: 79 II: 7 III: 5
Idiopathic osteonecrosis	8
Rheumatoid arthritis	1

**Fig. 3:**
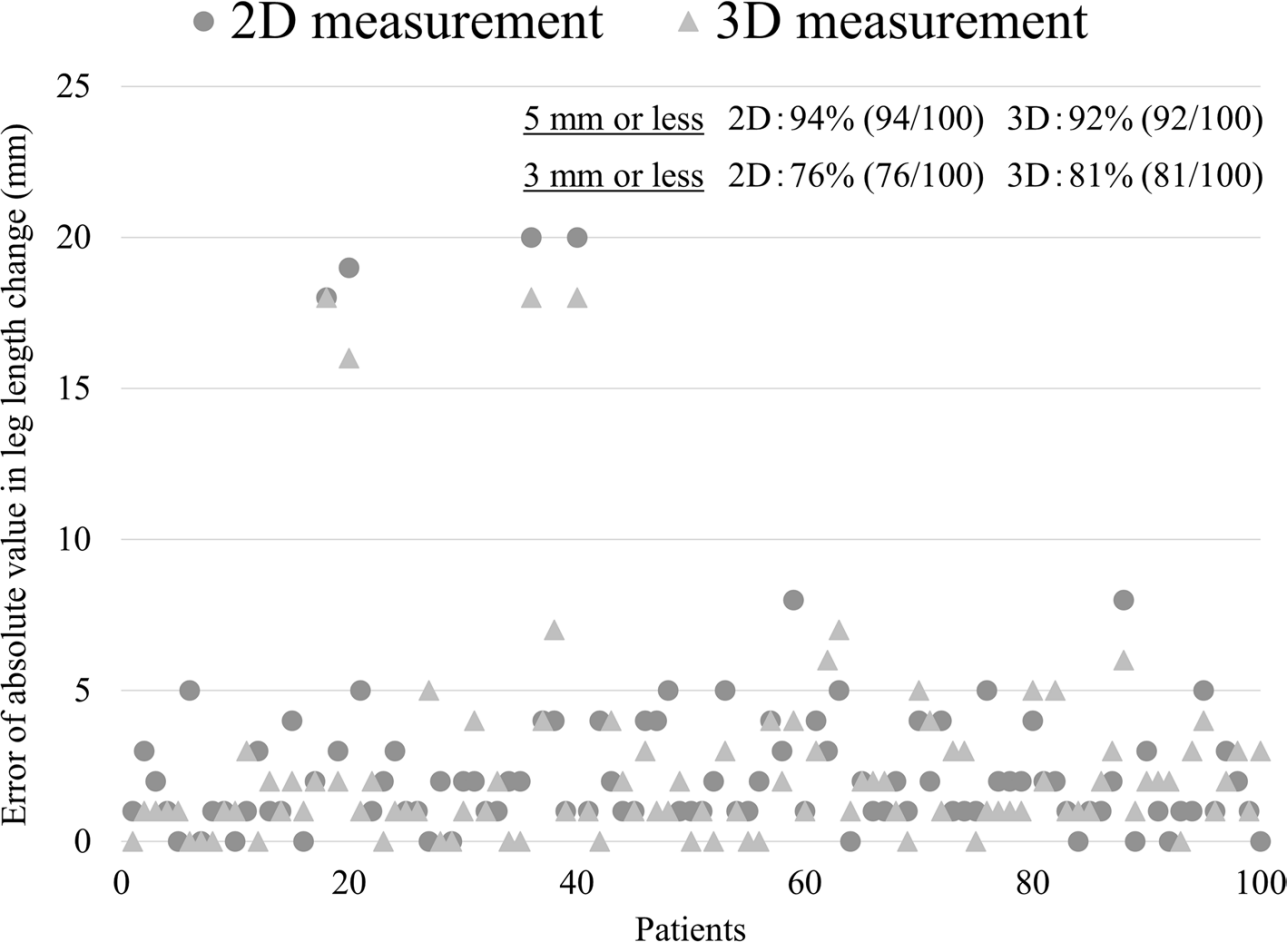
Dot diagram of the accuracy of image-free navigation based on 2D and 3D measurements

**Fig. 4:**
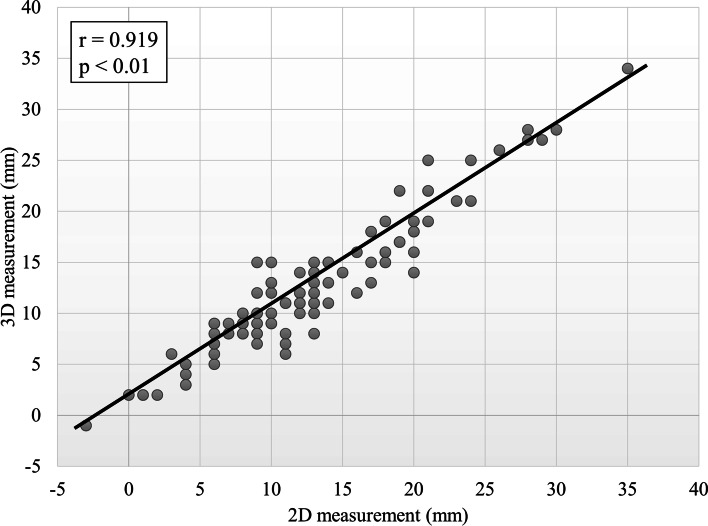
Correlation of leg length change between 2D and 3D measurements

Mean intraoperative LLC with image-free navigation was 12.0 ± 7.2 mm (range, −7 to 32 mm). Mean LLC on 2D measurement were 13.2 ± 7.0 mm (range, −3 to 35 mm). Mean LLC on 3D measurement were 12.9 ± 6.5 mm (range, −1 to 34 mm) (Table 2). Intraoperative LLC with image-free navigation was significantly shorter than LLC on 2D and 3D measurements. There was no significance between 2D and 3D measurements of LLC. In terms of the accuracy of image-free navigation based on 2D measurement, agreement with a difference ≤ 5 mm was confirmed in 94 of 100 THAs (94.0%), agreement with a difference ≤ 3 mm was confirmed in 76 of 100 THAs (76.0%). In the accuracy of image-free navigation based on 3D measurement, agreement with a difference ≤ 5 mm was confirmed in 92 of 100 THAs (92.0%), agreement with a difference ≤ 3 mm was confirmed in 81 of 100 THAs (81.0%) (Fig. 3). Errors in absolute value for LLC between 2D and 3D measurements were 1.7 ± 1.4 mm (range, 0 to 6 mm). A strong significant correlation between 2D and 3D measurements of LLC was observed (Fig. 4).

**Table 2:** Results of intraoperative leg length change with image-free navigation, leg length change on 2D measurement and leg length change on 3D measurement

	Image-free navigation	2D measurement	3D measurement
LLC (mm)	**12.0 ± 7.2**	**13.2 ± 7.0**	**12.9 ± 6.5**
	**(−7 to 32)**	**(−3 to 35)**	**(−1 to 34)**

## Discussion

THAs have very high success rates in terms of providing pain relief and improving mobility among patients with advanced osteoarthritis, osteonecrosis, and rheumatoid arthritis. However, these adult reconstructive procedures are also associated with a known potential for major complications, which may lead to litigation [22–24]. Although leg length discrepancy must be ≤10 mm for a patient to have good quality of life, an unexpected difference of 10–16 mm can sometimes occur despite careful attention [3, 6, 25]. Accurately assessing the amount of change in intraoperative leg length is considered very important to minimize the unexpected leg length discrepancy.

Good results have been reported for intraoperative LLC using image-free navigation in previous studies [13–15]. Moreover, several studies of intraoperative LLC have reported the accuracy of image-free navigation using pin fixing was significantly higher than free-hand, fluoroscopy and image-free navigation with the pinless device [26–28]. In the present study, good accuracy of image-free navigation in intraoperative LLC was confirmed on radiographic assessment, as in previous studies. The most important finding of the present study was the observation of the strong significant correlation of LLC between the evaluation of 2D and 3D measurements. Furthermore, no significant difference was observed the accuracy of LLC between the evaluation of 2D and 3D measurements. Based on these results, we were not confirmed the evaluation of 3D measurement was usefulness than that of 2D measurement in the present study.

On the other hand, a leg length error ≥ 10 mm was observed in 4 cases of this study. Ellapparadia et al. reported that 4 patients showed leg length error > 10 mm among these 6 patients with leg length error > 6 mm [13]. As the potential source of assessment errors in intraoperative assessment, loosening of the device has been reported as a factor inducing error [15]. It is thus necessary to keep in mind that some patients still show LLC >10 mm, although we were unable to clarify the causes of error in the present study.

Several limitations to this study must be considered. First, CT scans expose the patient to irradiation. CT scans is the imaging study that can be used for measurement of the hip geometry, preoperative planning and offset evaluation, but it exposes the patients to large radiation dose compared to conventional radiography [29–31]. Increased radiation exposure has been related to increased risk of various cancers, indicating the importance to minimize radiation exposure as much as possible [32, 33]. Therefore, a low dose CT have recently used to preoperative planning and postoperative assessment of total hip arthroplasty [34, 35]. However, we indicated that CT scan might not be necessary for the evaluation of leg length in this study. Second, leg length is often susceptible to errors that can be influenced by flexion contracture and variations in pelvic tilt and rotation. Fortunately, patients with severe flexion contracture were not observed in this study. A third limitation was the difference in evaluation methods between radiograph and CT examinations. 2D measurement was assessed as the distance between the horizontal line connecting both tear drops and the medial apex of the lesser trochanter, while 3D measurement was assessed as the distance from the ASIS to the intercondylar fossa of femur. However, we obtained the strong correlation between 2D and 3D measurement. From this result, the difference between 2D and 3D measurement was little affected for the evaluation of LLC.

## Conclusions

The accuracy of image-free navigation in leg length change showed good results for evaluations by both anteroposterior pelvic radiographs and 3D bone models using CT data. With regard to leg length change, evaluation using radiographs alone is possible if accurate radiographs can be obtained.

## Data Availability

The datasets used and analysed during the current study available from the corresponding author on reasonable request.

## Abbreviations

LLD: Leg length discrepancy
THA: Total hip arthroplasty
LLC: Leg length change
2D: Two-dimensional
3D: Three-dimensional
CT: Computed tomography
ASIS: Anterior superior iliac spine
